# Island Posterior Thigh Flap Revisited in Covering Extensive Sacral Wounds: Our Experience with Two Patients

**DOI:** 10.1155/2017/2084695

**Published:** 2017-02-22

**Authors:** F. Nangole Wanjala, Ajujo Martin

**Affiliations:** Department of Surgery, University of Nairobi, P.O. Box 2212, Nairobi 00202, Kenya

## Abstract

Deep sacral wounds are best covered by flaps. Posterior thigh flaps have routinely been used to cover such wounds. The flap can however be modified as an island flap. Two patients with extensive sacral wounds were managed with island posterior thigh flaps. Both patients were admitted secondary to road traffic accident with subsequent soft tissue loss of the sacral area. The sacral defects in both patients were approximately 17 cm by 23 cm in dimensions. Unilateral island posterior thigh flap was raised and used to cover the wounds. Postoperatively both patients did well; the donor site and recipient sites healed without any complications. Island posterior thigh flap is thus an option in covering extensive defects of the sacral area. The flap is reliable and easy to raise and has minimal donor site morbidity. By raising it as an island flap the dog ear defect is avoided and the flap is able to be tunneled under the gluteal muscle. This maneuver enables the flap to be advanced further allowing it to cover more distal and extensive defects.

## 1. Introduction

Sacral wounds require reconstruction with tissues that would allow them to withstand pressure during sitting or lying position. Adequate soft tissue padding with muscle or fasciocutaneous flaps provide the best options. A Variety of flaps have been described in literature [1–3]. Extensive sacral defects with involvement of the local tissue however make the majority of these flaps difficult to use and hence we need to use either regional, distant, or free flaps.

We present two patients with extensive sacral wounds reconstructed with island posterior thigh flaps.

## 2. Case  1

**Figure 1:**
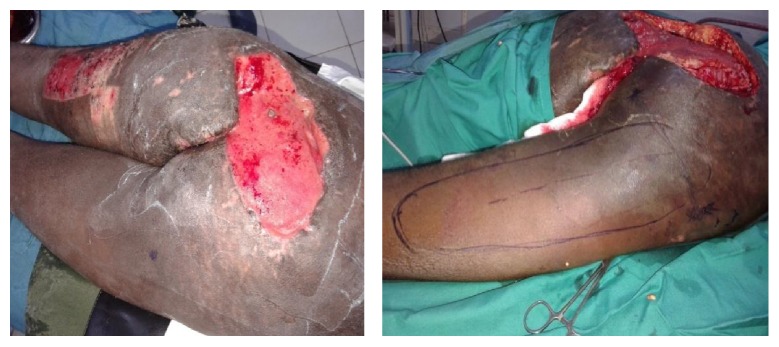
First patients with extensive sacral wounds and markings for the flap.

**Figure 2:**
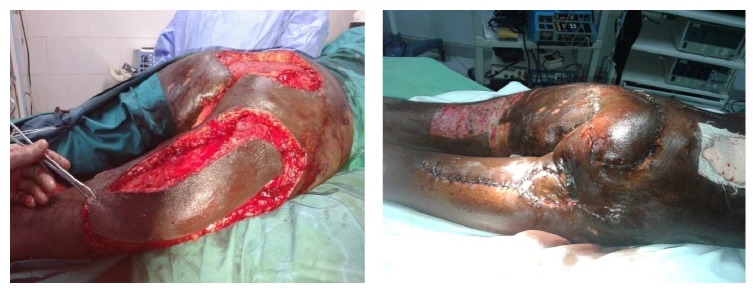
Island posterior thigh flap being raised in the first patient with no recurrence at three months of follow-up.

**Figure 3:**
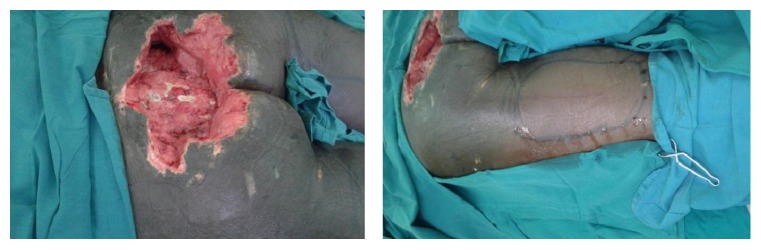
Extensive sacral wounds in the second patient with markings for the flap.

**Figure 4:**
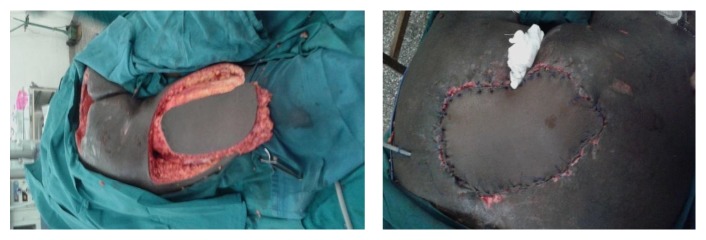
Flap being raised and inserted in the recipient site of the second patient.

A male patient aged 26 years was referred to the Plastic Surgical Department, Kenyatta National Hospital, with extensive injuries in the sacral area as a result of road traffic accident (Figure 1). The patient had sustained injuries secondary to a motor cycle injury. On examination the patient was in good general condition. He had also sustained fractures to the right tibia. He had no neurological deficits. After initial resuscitation and stabilization, the patient was booked for surgical debridement of both wounds. Repeat debridement was done on the sacral wounds until the wound was ready for closure. At surgery the defect measuring about 16 cm by 24 cm was encountered (Figure 1) An island posterior thigh flap based on the inferior branch of the inferior gluteal artery was raised (Figures 2, 3, and 4). The inferior gluteal vessels were identified with the aid of hand held Doppler. The inferior branch was noted to be in the midpoint between the ischial tuberosity and the greater trochanter. Markings for the flap were done along the axis of the vessels with dimensions measuring about 16 cm by 24 cm. The flap was raised distally first with the plane of dissection under the fascia l lata. It was then cut both medially and laterally. Proximally the flap was cut through the skin and subcutaneous tissue until the fascia and the gluteal muscle was identified. Once the pedicle was identified thinning of the flap was done while preserving the blood vessels. This was done up to the origin of the inferior gluteal vessels into the sacral promontory. The flap was then completely detached distally, medially, and laterally from all the surrounding tissues and tunneled under the gluteus muscle. The flap was then advanced into the wounds and closed in two layers over a portovac drain (Figure 2).

## 3. Case  2

**Figure 5:**
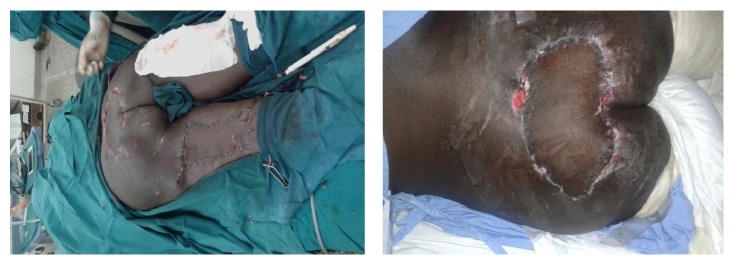
Skin graft of the donor site with the flap fully taken with no recurrence at one month after surgery.

A forty-year-old male patient was admitted at Kenyatta National Hospital with history of having sustained a road traffic accident while driving a car. The patient sustained injury to the spine with resultant paralysis of both lower limbs. He also sustained a crush injury to the sacral region with degloving injury of the superior gluteal muscles. The patient was initially resuscitated. Serial debridement was then done for sacral wounds and dressed with the negative pressure dressings. After about three weeks of admission, the patient was ready for wound closure. The defect measured about 14 cm by 20 cm in size. A left posterior thigh flap was fashioned as described above (Figures 3 and 4). The flap was completely detached and advanced into the wound underneath the gluteus muscle. A drain was inserted and the patient was managed in a prone position. The wounds were fully healed after about two weeks of surgery (Figure 5).

## 4. Discussions

Deep sacral wounds posttraumatic or as a result of pressure necrosis are best reconstructed with flaps. A variety of flaps have been described in the reconstruction of these wounds. These include transverse lumbar flaps, posterior thigh flaps and the gluteal muscle, or fasciocutaneous flaps [4–6]. Majority of the wounds can comfortably be covered by any of these flaps. However extensive wounds involving the sacral area and extending into the gluteus muscle like in our patients can be a challenge to reconstruct. In such wounds the gluteal muscle flap may not be an option since they may be involved in trauma. The transverse lumbar flap maybe limited by the extent of its reach and usually leaves the most distal aspect of the wound uncovered. The best option would thus be the posterior thigh flap.

Posterior thigh flap is a reliable flap that could be raised based either on the distal branch of the inferior gluteal vessels or on the posterior thigh perforators. With this flap most of the fasciocutaneous tissue on the posterior aspect of the thigh can be utilized to cover defects around the pelvic region. The flap has an added advantage of being sensate since the posterior nerve of the thigh could be incorporated into the flap. The flap could either be raised as an island flap or as a flap with skin in continuity with the gluteal tissues.

As an island flap, the posterior thigh flap has advantages over the nonisland flap. When raised as an island flap the arc of rotation for the flap is increased and the flap is thus able to reach further defects. The dog ear in the flap is also eliminated and this allows for the flap to be more malleable and hence ease of insertion into the recipient defect. The other advantage of the island flap is that it allows for the flap to be tunneled under the gluteus muscle and thus reduction of the distance is covered by the pedicle to the recipient site. This allows for flap tissue to cover more sacral wound.

In conclusion, extensive sacral wounds are best reconstructed with the island posterior thigh flap. The flap when raised as an island extends the reach of the flap and thus allows it to cover further and wider defects. The flap has an added advantage of bringing sensation in to the wound. It is also a reliable flap with robust blood supply that is easy to raise.
